# Recombinant human erythropoietin does not affect several microvascular parameters in well‐trained cyclists

**DOI:** 10.14814/phy2.13924

**Published:** 2018-12-27

**Authors:** Willem A. J. Birkhoff, Jules A. A. C. Heuberger, Titiaan E. Post, Pim Gal, Frederik E. Stuurman, Jacobus Burggraaf, Adam F. Cohen

**Affiliations:** ^1^ Centre for Human Drug Research Leiden The Netherlands; ^2^ Leiden University Medical Center Leiden The Netherlands; ^3^ Leiden Academic Centre for Drug Research Leiden The Netherlands

**Keywords:** Blood Doping, flowmetry, hematocrit, hyperemia, laser speckle contrast imaging, microcirculation

## Abstract

Recombinant human erythropoietin (rHuEPO) has been used as a performance‐enhancing agent by athletes in a variety of sports. The resulting increase in hematocrit levels leads to increased blood viscosity and can affect blood flow, potentially increasing the athlete's risk of developing health complications. However, the actual effects of using rHuEPO on microvascular blood flow and post‐occlusive reactive hyperemia are currently unknown. We therefore evaluated the effect of rHuEPO on the cutaneous microcirculation in well‐trained cyclists using laser speckle contrast imaging (LSCI). This study was part of a randomized, double‐blind, placebo‐controlled, parallel trial designed to investigate the effects of rHuEPO in 47 well‐trained adult cyclists (age 18–50 years). Subjects received a weekly dose of either rHuEPO or placebo for 8 weeks, and LSCI was performed at baseline, after a maximal exercise test in week 6, and before maximal exercise in week 8. Endpoints included basal blood flux, maximum post‐occlusion reperfusion, and time to return to baseline. Despite an increase in hematocrit levels in the rHuEPO‐treated group, we found no statistically significant difference in microvascular function measured between the rHuEPO‐treated group and the placebo group. Our results suggest that the increased hematocrit levels in rHuEPO‐treated well‐trained cyclists are not associated with changes in microvascular blood flow or post‐occlusive reactive hyperemia measured using LSCI.

## Introduction

The protein erythropoietin (EPO) is produced in the kidney and stimulates erythropoiesis in the bone marrow. EPO production is often greatly reduced in patients with end‐stage renal disease, leading to severe anemia. Recombinant human EPO (rHuEPO) is a biologically synthesized protein that helps maintain steady‐state erythropoiesis (Eschbach et al. 1989); in 1989, rHuEPO became available for clinical use in patients with chronic renal failure.

Despite conflicting evidence with respect to its efficacy, rHuEPO has been used as a performance‐enhancing agent by athletes in a variety of sports, including elite cycling. The use of rHuEPO by athletes is generally based on the notion that increasing hematocrit levels (i.e., increasing the ratio between the volume of red blood cells and total blood volume) increases the amount of oxygen available for the muscles, thereby increasing performance. However, there is currently no clear evidence to suggest that this is the case in elite cyclists (Heuberger et al. 2013).

In addition, extensive “doping” with rHuEPO has been reported with severe clinical outcomes in healthy athletes (Ramotar 1990; Lage et al. 2002). Although the risks associated with rHuEPO use have been well studied in patients with kidney disease (Robles 2016), little research has been performed regarding possible health effects in athletes.

An increase in hematocrit levels causes increased blood viscosity, thereby decreasing blood flow (Pries et al. 1992). This change in blood flow can in turn lead to thromboembolic events as is described by Virchows triad (Lowe 2003), which can cause severe organ damage (Kwaan and Wang 2003). Furthermore, decreased blood flow can limit the ability of oxygen‐transporting erythrocytes to reach the muscle tissue, thereby possibly—and paradoxically—reducing athletic performance (Hardeman et al. 2014).

Administering rHuEPO to patients with kidney disease increases E‐selectin and P‐selectin and endothelin levels, suggesting that rHuEPO may affect the endothelium, platelet function, and blood pressure (Carlini et al. 1993). However, whether rHuEPO causes a change in microcirculatory blood flow and/or post‐occlusive reactive hyperemia in athletes is currently unknown. Furthermore, if—and how—rHuEPO affects the ability of the microcirculation to adapt during and after maximal exercise is also unknown. Interestingly, a previous study found an impaired microvascular response in rowers after maximal exercise (Stupin et al. 2018); thus, the combination of exercise and use of rHuEPO might lead to increased health risk in athletes.

Changes in blood flow can be obtained by studying the microvasculature using a noninvasive technique such as laser speckle contrast imaging (LSCI). LSCI measures cutaneous microvascular blood flow by detecting the movement of laser‐illuminated circulating red blood cells in dermal capillaries and has been used in numerous studies to assess microvascular function (Pauling et al. 2015; Cracowski and Roustit 2016; Matheus et al. 2017). Importantly, LSCI measurements have low variability and high discriminating power, making LSCI a robust tool for use in clinical studies (Roustit et al. 2010; Tew et al. 2011).

Here, we used LSCI to measure the effect of rHuEPO on the cutaneous microcirculation in well‐trained cyclists. This study was part of a larger randomized, double‐blind, placebo‐controlled, parallel clinical trial in which we studied the effect of rHuEPO on cycling performance in well‐trained cyclists (Heuberger et al. 2017).

The purpose of this study was to evaluate the effect of rHuEPO on basal‐ and hyperemia‐induced cutaneous blood flow in resting state measured at week 0 and week 8. We also investigated postmaximal exercise responses between groups (performed at week 6).

## Materials and Methods

### Study population

The complete study protocol was published previously by Heuberger et al. (2017). In brief, the study included 47 well‐trained cyclists with a maximum power output at baseline of ≥4 W/kg, a normal electrocardiogram (ECG) during exercise, a hemoglobin level of 8.0–9.8 mmol/L, and a hematocrit level <48%. Subjects who were taking any medication that could cause a possible interaction with the study medications and/or the study assessments were excluded from the study. The subjects were instructed to continue their weekly athletic training schedule during the study.

### Study design

This was a randomized, double‐blind, placebo‐controlled, parallel trial conducted at the Centre for Human Drug Research (CHDR) in Leiden, the Netherlands. The study protocol was approved by the Medical Review and Ethics Committee in Assen, the Netherlands, and was performed in accordance with Dutch law regarding medical research. The trial was registered in the Dutch Trial Registry (number NTR5643).

Subjects received a weekly abdominal subcutaneous injection of rHuEPO (NeoRecormon, Roche, Basel, Switzerland) or placebo (0.9% NaCl) for 8 weeks. LSCI data were obtained at three time points. The first measurement was performed prior to the first dose while the subject was at rest (baseline). The second measurement was performed in after 6 weeks of rHuEPO treatment, 10–30 min after a maximal exercise test. The third measurement was performed in week 8 prior to injection of the 8th dose while the subject was at rest. Measurements in week 6 and week 8 were planned, because a maximal increase in hematocrit was expected.

On each measurement day, the subjects were instructed to abstain from the use of alcohol for 24 h prior to the visit and to abstain from using tobacco‐ or nicotine‐containing products for at least 2 h prior to the visit and until they were discharged from the clinical unit. All measurements were performed with the subject in a seated position, in a climate‐controlled room at 20–24°C following a 5‐min acclimatization period, at approximately the same time of day.

### Laboratory assessment

Prior to each dose, the hematocrit level was measured using a Heamatokrit 200 centrifuge (Hettich Benelux BV, Geldermalsen, the Netherlands), and hemoglobin was measured using a HemoCue Hb 201 + analyzer (Radiometer Benelux BV, Zoetermeer, the Netherlands) at CHDR. In addition, samples were also collected at 2‐week intervals and were assayed at the hematology laboratory at Leiden University Medical Center. All measurements took place prior to the administration of each dose, and the results of the measurements were accessible only to staff members who were not blinded to the study.

### Dosing

The dosing regimen used in this study has been reported previously (Heuberger et al. 2017). In brief, each subject received a weekly dose of either rHuEPO or placebo (saline). The rHuEPO group received a weekly injection of 5000–10,000 international units (IU) of rHuEPO with the goal of achieving a hemoglobin level 110–115% above baseline without exceeding a hematocrit level of 52 L/L.

### Laser speckle contrast imaging (LSCI)

LSCI was performed using a PeriCam PSI imager (Perimed AB, Järfälla, Sweden). Measurements were performed on a patch of skin surface approximately 30 cm^2^ on the ventral side of the subject's forearm. A beanbag was used to immobilize the arm in order to decrease movement artifacts. The laser was placed 15 cm above the skin surface, and cutaneous microcirculatory blood flux was measured continuously with 22 images per second. Basal flux was recorded for 5 minutes, after which the brachial artery was occluded for 5 min by inflating a pressure cuff placed around the upper arm to approximately 200 mmHg, which was above the systolic blood pressure for all subjects. After 5 min, the cuff was fully deflated, inducing a hyperemic response. The following study endpoints were measured: (1) basal flux, as an average over the last minute before occlusion (BF) in arbitrary units (AU); (2) maximal flux, as an average over 10 seconds during the peak (MF) in AU; (3) the ratio of MF to BF, yielding post‐occlusion reactive hyperemia (PORH), expressed as a percentage (%); (4) the time to reach maximal flux (measured as the time elapsed between the end of occlusion and maximal blood flux) in seconds; and (5) the time to return to baseline (measured as the time elapsed between maximal blood flux and the return to basal flux) in seconds. The data were analyzed using the PIMsoft software program (Perimed AB, Järfälla, Sweden).

### Maximal exercise test

The protocol for this test has been described in detail (Heuberger et al. 2017). In brief, subjects performed the maximal exercise test on a Monark LC4r ergometer (COSMED, Rome, Italy); during the test the workload was increased by 25 W every 5 min until the subject reached exhaustion. In week 6, LSCI measurements were performed approximately on average 15 min after the maximal exercise test, to avoid any movement artifacts during measurement. PORH was calculated 5 minutes after occlusion as described above.

### Statistical analysis

Repeated measures over the time points baseline, week 6 and week 8 were analyzed using a mixed model analysis of variance with treatment, time, and treatment by time as fixed factors, the participants as a random factor and—if available—the average prevalue as a covariate. The measures that were analyzed using the mixed model of analysis included basal flux, maximal flux, the time to return to baseline, and hematology values.

LSCI ratio maximal flux/basal flux and time to maximal flux were analyzed using a unpaired Student's *t*‐test for the versus the placebo group.

For the placebo group, the estimated intrasubject variability in LSCI measurements between study days was calculated for the two at‐rest measurements (i.e., baseline and week 8). For both the rHuEPO‐treated group and the placebo group, the minimal detectable effect size (MDES) of the LSCI endpoints was calculated as a combined measure of the effect size and the estimated variability, assuming a parallel comparison of 24 subjects.

All calculations were performed using SAS for Windows V9.4 (SAS Institute, Inc., Cary, NC, USA), or Prism V6.05 (GraphPad Software, La Jolla, CA, USA).

## Results

### Study population

Microvascular function was assessed in a total of 47 well‐trained cyclists who received either rHuEPO (*n* = 23) or placebo (saline; *n* = 24). The demographics and characteristics of the two groups are summarized in Table 1. All subjects in the study were healthy males with resting blood pressure and heart rate values within the normal range.

**Table 1:** Summary of the subjects’ demographics and clinical characteristics measured at baseline

	Placebo group (*n* = 24)	rHuEPO group (*n* = 23)	*P*‐value
Age in years	33.5 (20–50.0)	33.0 (22.0–48.0)	0.8645
Weight in kg	76.9 (9.4)	76.8 (9.0)	0.9904
Height in cm	186 (7)	186 (8)	0.8738
Mean arterial pressure in mmHg	92.1 (7.1)	94.7 (6.0)	0.1657
Hemoglobin in mmol/L	8.9 (0.5)	9.1 (0.5)	0.3097
Hematocrit in L/L	0.435 (0.394–0.469)	0.436 (0.387–0.469)	0.5537
P_max_ W/kg	4.34 (0.26)	4.35 (0.37)	0.65696
*V*_O2_ max in mL/kg per min	56.0 (4.1)	55.4 (5.1)	0.38772

Data are expressed as the median (range) or mean (SD).

### Hematocrit levels

**Figure 1:**
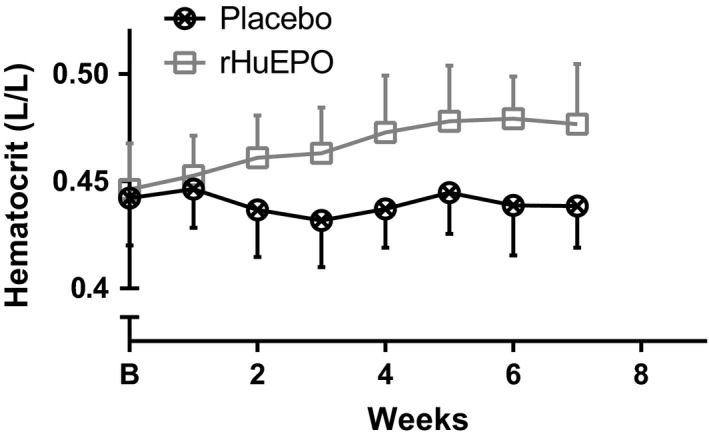
Time course of mean (±SD) hematocrit levels measured in the subjects who received placebo (*n* = 24) or rHuEPO (*n* = 23).

Figure 1 shows the hematocrit levels measured in the placebo and rHuEPO groups during the course of the study. As expected, the hematocrit levels increased significantly from 0.4334 L/L (95% CI: 0.4240, 0.4428) at baseline to 0.4757 L/L (95% CI: 0.4658, 0.4856) at week 7, in the subjects who received rHuEPO but was stable in the subjects who received placebo.

### Safety

Vital signs, including heart rate and blood pressure, were similar between the two groups.

### Variability and MDES

The intrasubject variability in subjects receiving placebo based on baseline and week 8 measurements of basal blood flow was (coefficient of variation [CV]) 14.4%, maximal flow 6.5%, and PORH 11.4%. The variability for the time to return to baseline and time to maximal flow was larger (CV: 29.2% and 22.8%, respectively).

Based on the calculated variances in the placebo group, MDES were calculated for the main microvascular measures, assuming a parallel study design with two groups of 24 subjects The MDES for LSCI basal blood flow was 5.21 AU, for maximal flow after occlusion 7.91 AU, and 50.97% for the effect of occlusion reperfusion. MDES for LSCI time to maximal flow was 3 sec, for time to return to baseline 23 sec.

### Contrast between the two groups

**Figure 2:**
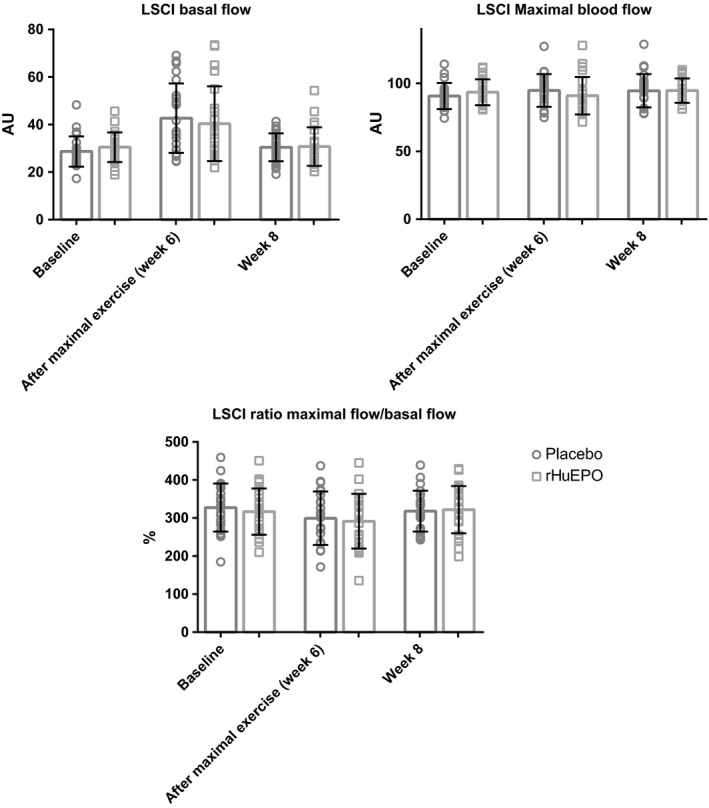
Summary of LSCI values measured in subjects who received placebo (*n* = 24) or rHuEPO (*n* = 23) at the indicated time points. The data are expressed as the mean values with SD.

Figure 2 summarizes the LSCI measurements between the rHuEPO and placebo groups. We found no significant difference between the rHuEPO and placebo groups with respect to basal blood flux (*P* = 0.97), maximal blood flux following occlusion (*P* = 0.41), or the ratio between peak flux and basal flux (*P* = 0.50). In addition, we found no significant difference between the two groups with respect to the time to reach peak flux or the time to return to basal flux (Table 2).

**Table 2:** Summary of LSCI kinetics measured at the indicated time points in subjects who received placebo (*n* = 24) or rHuEPO (*n* = 23)

LSCI parameter	Baseline (at rest)			6 weeks (after max exercise)			8 weeks (at rest)		
	Placebo	rHuEPO	*P*‐value	Placebo	rHuEPO	*P*‐value	Placebo	rHuEPO	*P*‐value
Time to maximal flux (sec)	11.92 (4.19)	10.61 (3.43)	0.2491	11.17 (6.34)	10.00 (6.91)	0.5494	11.63 (3.98)	11.48 (5.41)	0.9158
Time to return to basal flux (sec)	125.5 (32.6)	112.2 (23.2)	0.1158	135.7 (35.0)	153.3 (47.4)	0.1515	136.6 (28.7)	127.5 (29.5)	0.2913

Data are expressed as the mean (SD).

## Discussion

Here, we used LSCI to study the effect of rHuEPO injections and maximal exercise on the microvasculature and post‐occlusive reactive hyperemia in well‐trained cyclists. As expected, rHuEPO caused a significant increase in hematocrit levels; in contrast, however, we found no difference between the rHuEPO and placebo groups at 8 weeks with respect to blood flow in the cutaneous microvasculature. In addition, we found no difference between the two groups with respect to maximal flux in week 6 after the subjects performed the maximal exercise test.

LSCI is a highly sensitive method for measuring acute microvascular changes in response to various challenges such as an inspiratory breath hold, local thermal hyperemia, and post‐occlusion hyperemia (Roustit et al. 2010; Birkhoff et al. 2018). Previous studies using LSCI found that patients with sickle cell disease have increased basal microvascular blood flux possibly due to decreased hematocrit levels (Birkhoff et al. 2018). In contrast, patients with polycythemia vera have increased hematocrit levels and decreased microvascular blood flow(Kwaan and Wang 2003); this decreased blood flow in these patients can lead to pathological conditions such as hypertension, thromboembolism, stroke, and/or other severe clinical complications. However, the changes compared to healthy individuals in hematocrit levels measured in these patients were two‐ to threefold larger than in the subjects in our study who received rHuEPO.

In our analysis, we found intermediate variability with respect to our LSCI measurements, with a CV ranging from 7% to 14% (data not shown); this variability is slightly higher than previous reports (with CV values of 6–10%) (Roustit et al. 2010; Birkhoff et al. 2018). Importantly, the previous studies measured variation over a 1‐week period, compared to 8 weeks in our study; thus, the longer time frame in our study may have contributed to the slightly higher variability. An additional source of variability may be due to the physiology of the well‐trained cyclists in our study. However, any clinically relevant changes would have been observed by LSCI measurements, which are shown by the calculated MDES of 5.21 AU for basal blood flux which was small and below relevant physiological changes.

Based on Poiseuille's law, the classic view is that a change in hematocrit levels can affect the blood's viscosity, thereby affecting blood flow (Pirofsky 1953; Baskurt and Meiselman 2003). However, recent studies suggest that a relatively small change in the hematocrit level of up to 13% may not directly affect microvascular blood flow (Martini et al. 2005) or cardiac output in healthy volunteers (Lundby et al. 2008). Thus, the effect of a change in the hematocrit level on blood flow may have a threshold and may only become evident with a relatively large change in hematocrit levels over a longer period of time. This notion is supported by our measurements of blood pressure, which were unaffected in the rHuEPO group. Accommodating a relatively small change in hematocrit levels over a short period can be compensated for by vascular autoregulatory mechanisms such as dilation of the vessels. However, this can only occur if there is sufficient autoregulatory capacity in the vessels (Baskurt and Meiselman 2003), which might not be the case for patients with sickle cell disease or polycythemia vera.

Although no difference was observed in LSCI PORH measurements due to rHuEPO, nor after maximal exercise challenge, a possible effect on the endothelium was observed in other markers: P‐selectin and E‐selectin (Heuberger et al. 2017). These findings can be indicative that short‐term exposure to rHuEPO has an effect on platelets and/or the endothelium, but does not affect the reactive capability of the endothelium. However, other factors, such as dehydration, exhaustion and extreme temperatures, might also influence the hematocrit levels due to fluid shifts or loss and thereby the microcirculatory flow and endothelial function in elite cyclists during races (Trangmar and González‐Alonso 2017).

This is the first reported placebo‐controlled study of the effect of rHuEPO—and indirectly the accompanying changes in hematocrit levels—on microvascular blood flow in well‐trained athletes. Although previous studies investigated the effect of exercise and rHuEPO on post‐occlusion hyperemia in healthy volunteers, this is the first study to closely mimic the reported situation in professional cycling, in which rHuEPO is used as a doping agent. As we did not observe any difference in microvascular flow, it is unlikely that the use of rHuEPO has negatively influenced the athletic performance by limiting the oxygen‐transporting capabilities of the erythrocytes. This is also reflected in the exercise performance results published elsewhere (Heuberger et al. 2017).

A possible limitation of our study is that the subjects in the rHuEPO group had a relatively small—albeit significant—increase in hematocrit levels. We therefore cannot exclude the possibility that a larger increase in hematocrit levels (e.g., in professional cyclists who doped with rHuEPO in the past) may affect microvascular function. Because of the use of the Athletes Biological Passport and stringent doping controls, the increase in hematocrit levels observed in this study are most likely similar or even higher as what they would be in elite cyclist currently using rHuEPO as doping.

In summary, we report that weekly injections of rHuEPO increases hematocrit levels in well‐trained cyclists, but does not appear to affect either pre‐exercise or postexercise microvascular blood flow measured using LSCI.

## Conflict of Interest

None declared.
